# Chromon-3-aldehyde derivatives restore mitochondrial function in rat cerebral ischemia

**DOI:** 10.22038/ijbms.2020.46369.10710

**Published:** 2020-09

**Authors:** Dmitry I. Pozdnyakov, Andrey V. Voronkov, Viktoriya M Rukovitsyna

**Affiliations:** 1 Department of Pharmacology Pyatigorsk Medical Pharmaceutical Institute, Pyatigorsk, Russia; 2 Department of Organic Chemistry, Pyatigorsk Medical Pharmaceutical Institute, Pyatigorsk, Russia

**Keywords:** Acetylcysteine, Apoptosis, Brain ischemia, Cell respiration, Laboratory animals, Mitochondria

## Abstract

**Objective(s):**

This work aimed to assess the effect of 10 new chromon-3-aldehyde derivatives on changes of mitochondrial function under the conditions of brain ischemia in rats.

**Materials and Methods:**

The work was executed on BALB/c male-mice (acute toxicity was evaluated) and male Wistar rats, which were used to model cerebral ischemia by permanent middle cerebral artery occlusion. The test-substances, 10 derivatives of chromon-3-aldehyde and the reference drug, N-acetylcysteine, were injected after modeling of ischemia for 3 days. After that, neurological symptoms, the area of cerebral infarction, and change in mitochondrial function were evaluated.

**Results:**

It was established that use of all chromon-3-aldehyde derivatives contributed to the recovery of mitochondrial function, which was reflected in enhanced ATP-generating activity, maximum respiration level, respiratory capacity, as well as reduction in the intensity of anaerobic reactions, apoptosis, and normalization of the mitochondrial membrane potential. The most pronounced changes were noted with the use of 6-acetyl substituted chromon-3-aldehyde derivative, the administration of which decreased neurological symptoms and size of brain necrosis area.

**Conclusion:**

The obtained data may indicate the most pronounced neurotropic effect in a number of test-objects has the 6-acetyl substituted derivative of chromon-3 aldehyde, realized by restoration of mitochondrial function, which may be the basis for further study of chromon-3-aldehyde derivatives.

## Introduction

Stroke is the most common angioneurological disease, which is one of the leading causes of primary disability and mortality in worldwide (1). Two main forms of stroke are known: ischemic and hemorrhagic. The first pathogenetic variant of stroke is associated with the occlusion of cerebral vessels with a thrombus or embolus with the formation of a necrotic area - a zone of cerebral infarction. Hemorrhagic form of stroke, mediated by rupture of cerebral vessels and intracerebral hemorrhage (2). As a rule, most cases (70-80%) of a stroke occur in its ischemic variant, which has therapeutic strategies that are fundamentally different from a hemorrhagic stroke. Over the past decades, significant progress has been achieved in understanding the pathophysiology of stroke: from substantiating the concept of «ischemic penumbra» to understanding spontaneous thrombolytic processes and the phenomenon of ischemia-reperfusion (3). 

The primary factor for the classic «ischemic cascade” of brain damage is the limitation of cerebral blood flow, which triggers a number of pathogenetic events leading to neuronal death and an increase in the necrosis area of brain tissue, which in turn mediates the appearance of specific neurological and cognitive symptoms of ischemic stroke (4). Among the data on the pathophysiological basis of cerebral ischemia, a violation of the functional activity of mitochondrial neurons can be attributed (5). Mitochondria are dynamic cellular organelles involved in the processes of cellular respiration (provide synthesis of macroergic compounds), regulation of apoptotic and redox cellular reactions (6). Damage to mitochondria plays a critical role in the initiation of oxidative stress, the intensification of both the internal and external apoptosis pathways, as well as the activation of anaerobic processes leading to an increase of the cerebral infarction area (7). To date, it has been established that mitochondria are the main sources of reactive oxygen species (ROS) in the cell, and their (ROS) overproduction is noted both under conditions of oxygen deficiency and during reperfusion, when tissue oxygenation increases significantly (8). Transfer of electrons along the mitochondrial respiratory chain at the level of complexes I and II leads to an increase in the formation of superoxide anion radical, which directly or indirectly (through an increase in the activity of protein kinase C and MAPK) enhances processes of cell structure oxidation and damage (9). At the same time, a decrease in the mitochondrial membrane potential, which is observed when the electron transport is uncoupled along the electron transfer chain, leads to a decrease in the proton gradient and, as a consequence, a decrease in the function of F_1_F_0 _ATP synthase, which significantly affects the production of macroergic phosphates in the ATP form (10). Under current conditions (a decrease in the synthesis of ATP and mitochondrial membrane potential), activation of proapoptotic signaling pathways is noted, the earliest and most unfavorable of which is the formation of mitochondrial pores of transition permeability (MPTR) (11). As a result of the opening of MPTP, there is an increase in cytochrome C releasing from the mitochondrial matrix, which in the cytosol interacts with the factor activating apoptotic protease (Apaf-1), and this factor in combination with deoxyadenosine triphosphate, activates caspase-9. Therefore of this reactions cascade, caspase-9 dephosphorylates caspase-3, which sequences DNA fragments (12). The second, mitochondrial-dependent, proapoptotic mechanism is the pathway triggered by apoptosis-inducing factor (AIF). Mitochondrial protein AIF is defined as the main caspase-independent regulator of apoptosis, which, after translocation to the nucleus, inhibits poly-ADP ribosyl polymerase, thereby enhancing and accelerating the apoptotic process (13). It should be noted that both caspase-dependent and caspase-independent apoptotic events occur in the cell almost simultaneously, and the primary trigger of these processes is a violation of the mitochondria function and structure (14).

Thus, based on the significant role of mitochondrial dysfunction in the pathogenetic cascade of ischemic brain damage, the advisability of pharmacological correction of this pathophysiological state, as one of the new directions of cerebroprotective therapy сan be assumed. Chromon derivatives are biologically active compounds of natural or semi-synthetic origin that have a broad spectrum of pharmacological activity, including antioxidant (15), antibacterial, anti-inflammatory, insecticidal (16), hypoglycemic (17), and antiviral properties (18). It is also known that chromon derivatives prevent aggregation of β-amyloid, inhibit the activity of monoamine oxidase, which can have a positive effect on the course of Alzheimer’s disease (19). In this regard, we can assume the relevance of the study of new chromon derivatives, in order to expand the spectrum of pharmacological activity. These compounds include derivatives of chromon-3-aldehyde.

In previous studies, we have found a positive effect of some chromon-3-aldehyde derivatives on the course of apoptotic reactions triggered by AIF (20), as well as on the development of oxidative stress (21). Thus, it was found that the use of chromon-3-aldehyde derivatives reduced the concentration of apoptosis-inducing factor in ischemic tissue, and also contributed to an increase in the concentration of ATP and the activity of endogenous antioxidant enzymes: superoxide dismutase, glutathione peroxidase, and catalase. However, other aspects of the chromon-3-aldehyde derivatives influence on the change in mitochondrial function may be of interest, which served as the aim of this study.

The aim of the study was to evaluate the effect of certain derivatives of chromon-3-aldehyde on changes in the activity of neuron mitochondria under cerebral ischemia conditions in rats.

## Materials and Methods

***Experimental animals***

The study was performed on 95 male-mice of the BALB/c line weighing 20-25 grams and 260 male rats of the Wistar line weighing 210-230 grams. The animals were obtained from the «Rappolovo» laboratory animal nursery and were kept under standard conditions of living systems laboratory at the Pyatigorsk Medical and Pharmaceutical Institute. Before being included in the work, the animals underwent microbiological control and two-week quarantine. Conditions of detention: animals were housed by 5 individuals in macrolon cages of the T-2 (rat) and T-3 (mouse) type with a natural change in light-dark mode and relative humidity of 60±5%, ambient temperature of 20±3 °C. Extruded feed, balanced by nutrient content, and water, animals received *ad libitum*. In planning and carrying out the study, the authors were guided by generally accepted norms of experimental ethics (Directive 2010/63 / EU of the European Parliament and of the council on the protection of animals used for scientific purposes, September 22, 2010). The concept of this work was approved by the local ethics committee (protocol No. 1 of 10/30/2019).

***Test compounds***

Chromon-3-aldehyde derivatives (Table 1) synthesized at the Department of Organic Chemistry of the Pyatigorsk Medical and Pharmaceutical Institute using the well-known reaction with Vilsmeier’s reagent were used as the test-objects in this study (22). The structure and physicochemical properties of the test-substances were confirmed by NMR spectroscopy (Appendix 1). The studied compounds were substituted derivatives of chromon-3-aldehyde with the inclusion of various functional groups in the linker structure of the chromon: halogens, acetyl, methoxyl and aminomethyl phenyl groups. Also, the test-substances under the ciphers CB-2, CB-7 and CB-8 were oximes of the original connections. When performing the study, N-acetylcysteine at a dose of 150 mg/kg *per os* used as a reference drug (23).

***Study design***

**Figure 1:**
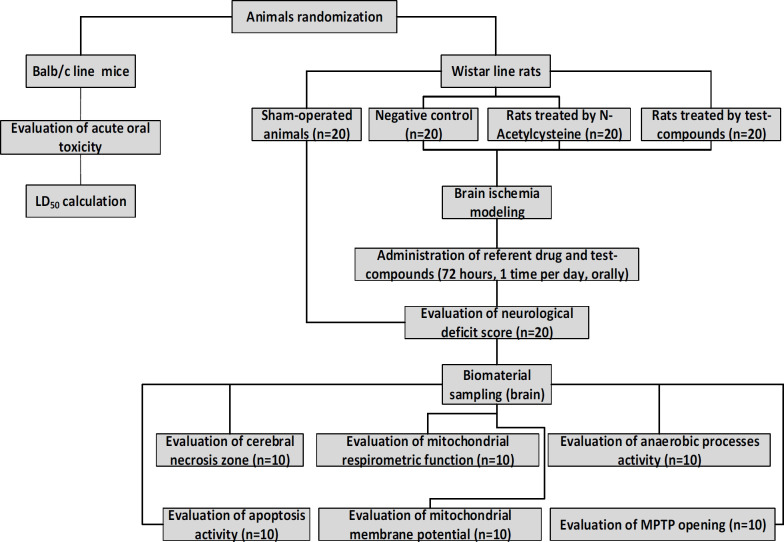
Study design

This work was performed in 2 stages (Figure 1). At the first stage, the «acute toxicity» of the test compounds with the determination of LD_50_ was evaluated. At the second stage of the experimental work, the effect of chromon-3-aldehyde derivatives on the change in the mitochondrial function under experimental cerebral ischemia was evaluated. The acute toxicity of the test substances was evaluated on BALB/c male mice. At the stage of evaluation the influence of the test objects on the change in mitochondrial function, Wistar male rats were used as a biological model, and the following experimental groups were formed (n=20 each group): a group of sham-operated rats (SO); negative control group (NC) - with cerebral ischemia, but without pharmacological support; a group of animals receiving a reference drug; and groups of rats treated by the test compounds (20 animals were taken to study one substance). The reference drug and the test-objects (at a dose of 1/100 from LD_50_) were administered *per os* through an atraumatic catheter 30 min after the reproduction of cerebral ischemia and then once a day for three days. On the 4^th ^day of the study, in rats assessed the degree of neurological deficit, after which the animals were decapitated under chloral hydrate anesthesia (350 mg/kg, intraperitoneally) and biomaterial was collected (brain). Then, mitochondrial function (n=10) and brain necrosis area was evaluated (n=10).

***Evaluation of «acute toxicity» of the test-compounds***

The study of the toxicity of chromon-3-aldehyde derivatives was carried out using the available method for studying the oral toxicity of chemical substances in an acute experiment - the «Up and down» procedure. According to this protocol, two tests were conducted: limit testing and basic testing. Since for some derivatives of chromon-3-aldehyde a rather low toxicity is described, in the conditions for determining «acute toxicity», we chose a limit dose of 5000 mg/kg, *per os* (14). The analysis and the LD_50_ calculation corresponded to the recommendations set out in OECD No. 425.

According to OECD recommendations No. 425 the range of administered doses for the test-compounds was: 1,75; 5,5; 17,5; 55; 175; 550; 1750; 5000 mg/kg with a dose progression coefficient of 3.2. The administration of the compounds was carried out fractionally with 1 hour time interval until the required dose was reached according to the following scheme: the first animal receives the test-objects at a dose of 1.75 mg/kg, if the animal survives, the administration of the compound is continued in increasing order of doses until the 1st case of death is reached. After registering a fatal outcome, the next animal receives a dose of the test-compounds an order of magnitude less than the previous one. The criteria for stopping the experiment were:

consecutive deaths of 3 animals;

inverting the administered dose 3 times in 4 consecutive animals;

no death of 4 consecutive animals when administered one dose, after reaching a fatal outcome.

*Cerebral ischemia model*

Brain ischemia was reproduced by *Tamura* method. In this study, irreversible coagulation of the right middle cerebral artery was used. Operation progress: animals were anesthetized by chloral hydrate (350 mg/kg, intraperitoneally). The area to the right of the eye was depilated, an incision was made and the soft tissues were moved apart, exposing the process of the zygomatic bone, which was removed. Next step, a trepanation hole was made and thermocoagulation of the middle cerebral artery was performed under the point of artery intersection with the olfactory tract. Later, as far as possible, the structure of soft tissues was restored. The suture was treated by an antiseptic solution (Benzyldimethyl [3-myristoilamine) propyl] ammonium chloride monohydrate); 0.01% solution) and the animals were left under a warming lamp until awakened (24). 

***Neurological deficit determination***

The degree of development of neurological deficit was evaluated according to the *McGraw* scale (1976). Evaluated parameters presented in Table 2.

The total score of 0.5-2.0 corresponded to a mild degree of neurological deficit; 2.5-5.0 moderate severity; 5.5-10 severe neurological deficit, while the degree of neurological deficit was determined by the sum of the relevant points (25).

***Biomaterial sample preparation***

The brain of experimental animals was used as biomaterial in the work. Anesthetized rats (chloral hydrate 350 mg/kg, intraperitoneally) were decapitated, after which the cranium was opened, the brain was removed (n=10), which was homogenized in a Potter mechanical homogenizer with media: 1 mmol EDTA, 215 mmol mannitol, 75 mmol sucrose, 0.1% BSA solution, 20 mmol HEPES, with a pH of 7.2 with a ratio of brain mass and buffer solution volume of 1: 7. The resulting homogenate was divided into two parts. The first was centrifuged in mode 5 min 10000g, while the brain tissue supernatant was removed for ELISA. The second part of the brain homogenate was used to assess the state of mitochondrial function, for which the homogenate was centrifuged in the mode of 1.400g → 3 min. at 4 °C, after which the supernatant was transferred to 2 ml tubes. Next, the resulting supernatant was centrifuged at 13000 g → 10 min and the supernatant (culture contains native mitochondria) was removed for analysis (20). In addition, in 10 rats, the brain was removed from the group, the cerebellum was cut off, the hemispheres were separated, weighed to the 0.001 g accuracy, and individually homogenized in 0.01 mmol/l PBS in a 1: 7 ratio. The resulting homogenate was removed to determine the size of the necrosis zone.

***Determination of the brain necrosis area***

Homogenate of the cerebral hemispheres were placed in cups with 10 ml of a 1% solution of triphenyltetrazolium chloride in phosphate buffer (pH 7.4). Sample bottles were placed in a water bath for 20 min at 37 °C. Next, this mixture was centrifuged at 5000 RPM / 10 min. The supernatant was removed and 3 ml of cooled chloroform were added to the precipitate. Shake for 2 min. Chloroform formazan extract was obtained for 15 min at 4 °С, shaking the mixture every 5 min for 30 sec. Centrifuged and measured optical density (492 nm) against pure chloroform. The calculation of the necrosis zone was expressed as a percentage of the total mass of the hemispheres:

$\overset{\text{\textasciitilde{}}}{o}=100-\frac{\mathrm{\varepsilon 1}\overset{\text{˙}}{I}1+\mathrm{\varepsilon 1}\overset{\text{˙}}{I}2}{\mathrm{\varepsilon 1}\overset{\text{˙}}{(\mathrm{I 1}+}\overset{\text{˙}}{I}2)}100$

where *x* is the size of the zone of necrosis as a percentage of the total mass of the brain;

ε_1_ is the optical density of the sample with an intact hemisphere;

ε_2_ is the optical density of the sample with a damaged hemisphere;

M_1_ is the mass of the intact hemisphere; 

M_2_ is the mass of the damaged hemisphere (26).

***Respirometric analysis***

Analysis of the mitochondria respiratory function was carried on the AKPM1-01L laboratory respirometer system (Alfa Bassens, Russia). The mitochondrial respiratory function was assessed by the change in oxygen consumption in the medium against the introduction of mitochondrial respiratory uncouplers. The last in the work were: oligomycin 1 µg / ml; 4 - (trifluoromethoxy) phenyl) hydrazono) malononitrile (FCCP-1 µM); rotenone - 1 µM; sodium azide - 20 mmol. ATP-generating ability was determined (by the difference in oxygen consumption after the addition of FCCP and oligomycin); the maximum level of respiration (according to the difference in oxygen consumption after the addition of FCCP and rotenone) and the respiratory capacity (according to the difference in oxygen consumption after the addition of FCCP and the basal level of oxygen consumption). The activity of glycolysis processes was evaluated when glucose (15 mmol/l) was used as an oxidation substrate The intensity of glycolysis was determined (according to the difference in oxygen consumption after adding glucose and the basal level of oxygen consumption), glycolytic capacity (according to the difference in oxygen consumption after adding oligomycin and glucose) and glycolytic reserve (according to the difference in oxygen consumption after adding glucose and sodium azide). During the analysis, the biosample volume was 275 μl, and 25 μl of injected analyzers. Oxygen consumption was determined in ppm with followed conversion to protein concentration in the sample. The protein content was determined by the Bradford method (27, 28). 

***The evaluation of the mitochondrial pore of transition permeability (MPTP) opening time***

The latent opening time of the mitochondrial pore was estimated by spectrophotometric method. The incubation medium contained: 0.5 ml of the analyzed supernatant, 200 mM KCl, 0.5 ml of a 1 μm solution of cyclosporin A. The resulting mixture was adjusted to 2 ml with HEPES buffer solution with a pH of 7.4. The absorbance of the mixture was recorded at λ=540 nm, then the resulting solution was incubated for 25 min at the room temperature and with constant stirring, and the absorbance was re-recorded in dynamics. The latent opening time of the mitochondrial pore was evaluated in seconds, recording a decrease in the extinction of the samples from 0.4 to 0.2 (29).

***The evaluation of mitochondrial membrane potential***

Mitochondrial membrane potential was evaluated by spectrophotometric method. The incubation medium contained: 0.5 ml of the analyzed supernatant, 0.5 ml of 9 μm solution of safranin O. The resulting mixture was adjusted to 2 ml with HEPES solution with a pH of 7.4. The absorbance of the mixture was recorded at λ=515 nm and λ=525 nm. The transmembrane electrochemical gradient, ∆Ψ was determined by the difference in absorbance: ∆Ψ = A_515_-A_525_. * 1000. (29).

***ELISA-study***

In this work, the concentration of caspase-3 and apoptosis-inducing factor (AIF) was determined by enzyme-linked immunosorbent assay in the supernatant of the animal’s brain. Species-specific ELISA kits manufactured by *Cloud clone corp*. (USA) were used in this block of experimental work. The course of the analysis corresponded to the manufacturer’s instructions attached to each set. Recording results on an *Infinite F50 *microplate reader (*Tecan, Austria*).

***Statistical analysis***

Statistical analisys of the obtained data was carried out in the software application package of statistical analysis STATISTICA 6.0 (StatSoft, USA). The results were expressed as M (mean)±SEM. Comparison of the mean groups was carried out by the analysis of variance method (ANOVA) with the Newman-Keuls* post-hoc* test, with a significance level of *P*<0.05.

## Results

***Evaluation of «acute toxicity» of the test-compounds***

In the course of an evaluation of the acute oral toxicity (Table 3) of the test chromon-3-aldehyde derivatives, it was found that none of the studied substances at a dose of 5000 mg/kg passed the limiting test, which implied a basic test. So, when setting up the main testing, it was noted that after the direct administration of the test substances, no animal deaths were noted. Further, during the 14-day observation period, a total of 36 mice of BALB/c males died: with the administration of compound CB-1, 3 individuals; CB-2 - 4 individuals; CB-3 - 3 individuals; CB-4 - 3 individuals; CB-5 - 2 individuals; CB-6 - 4 individuals; CB-7 - 4 individuals; CB-8 - 5 individuals; CB-9 - 3 individuals and CB-10 - 2 individuals. Based on the obtained results LD_50_ was calculated by the maximum likelihood method (30).

Thus, the LD_50 _value allows us to attribute all test derivatives of chromon-3-aldehyde to the 5^th^ chemical hazard class according to the GSH classification (31). Experimental doses of the test substances for further research are presented in Table 3.

***The effect of chromon-3-aldehyde derivatives on changes in neurological symptoms in rats under conditions of cerebral ischemia***

**Figure 2:**
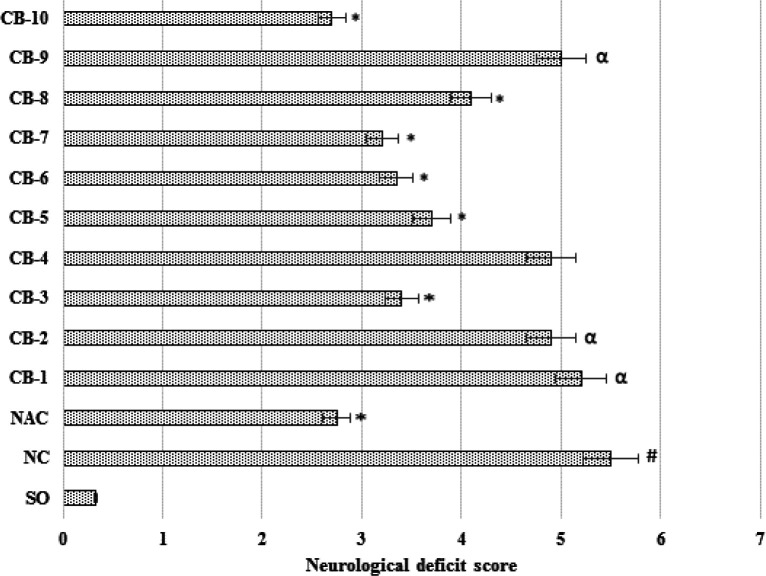
The effect of the test derivatives of chromon-3-aldehyde on the change of neurological deficit in rats under conditions of cerebral ischemia

Note: SO: sham-operated animals, NC: negative control rats, NAC: N-acetylcysteine treated rats, CB-1-10: rats that are treated by test compounds, # - statistically reliable respecting the SO group of animals, * : statistically reliable respecting the NC of the rat group, α : statistically significant relative to the group of animals treated by N-acetylcysteine

In assessing the degree of neurological deficit in rats with reproduced cerebral ischemia, it was found that in the NC group of animals the total score of neurological symptoms was 5.5±0.124 conventional units, which was 16.7 (*P*<0.05) times higher a similar indicator of the SO group of animals (Figure 2). The administration of the reference drug reduced neurological deficit in rats by 50% (*P*<0.05). Against the background of the administration of the test-compounds under the codes of CB-3; CB -5; CB -6; CB -7; CB -8 and CB -10, the total score of neurological deficit was lower than in the NC group of rats by 38.2%(*P*<0.05); 32.7% (*P*<0.05); 39.1% (*P*<0.05); 41.8% (*P*<0.05); 25.4% (*P*<0.05) and 50.9% (*P*<0.05), respectively (Figure 2). It should be noted that the use of chromon-3-aldehyde derivatives under laboratory codes CB-1; CB-2; CB-4 and CB-9 didn’t significantly affect on the change of neurological status of animals under conditions of cerebral ischemia.

***The effect of chromon-3-aldehyde derivatives on the change of the brain necrosis zone area in rats under cerebral ischemia***

**Figure 3:**
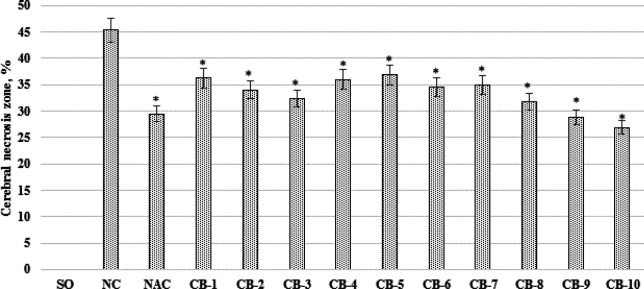
The effect of the studied derivatives of chromon-3-aldehyde on the change of the cerebral infarction area in rats under cerebral ischemia

Note: SO: sham-operated animals, NC: negative control rats, NAC: N-acetylcysteine treated rats, CB-1-10: rats that are treated by test compounds, # - statistically reliable respecting the SO group of animals, * : statistically reliable respecting the NC of the rat group, α : statistically significant relative to the group of animals treated by N-acetylcysteine

When conducting this block of experimental work, it was found that in the NC group of animals, the zone of cerebral infarction formed as a result of occlusion of the middle cerebral artery was 45.3±7.026% (Figure 3). The use of N-acetylcysteine contributed to a decrease in the cerebral necrosis zone by 35.1% (*P*<0.05) relative to the NC group of animals. At the same time, in rats treated by the test derivatives of chromon-3-aldehyde under laboratory codes CB-1; CB-2; CB-4; CB-5; CB-6 and CB-7, the value of the zone of cerebral infarction varied in almost the same range, namely, a decrease in the area of brain necrosis was observed in animals treated by these compounds, which was statistically significantly lower than the similar parameter of the NC of the rat group by 20% (*P*<0.05); 25% (*P*<0.05); 20.5% (*P*<0.05); 18.6% (*P*<0.05); 23.8% (*P*<0.05) and 22.9% (*P*<0.05), respectively. It is worth noting that the use of compounds CB-3; CB-8; CB-9 and CB-10 under conditions of cerebral ischemia contributed to a more pronounced decrease in the area of cerebral infarction, the value of which was 28.5% (*P*<0.05)% 29% (*P*<0.05) 36.5% (*P*<0.05) and 40.6% (*P*<0.05), respectively, less than that of the NC group of rats (Figure 3).

***The effect of chromon-3-aldehyde derivatives on the change in the respirometric function of the neuronal mitochondria in cerebral ischemia conditions***

**Figure 4:**
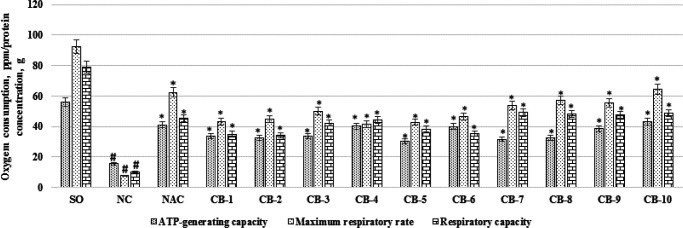
The effect of the test derivatives of chromon-3-aldehyde on the change in the overall respirometric function of the neuronal mitochondria under cerebral ischemia in rats

Note: SO: sham-operated animals, NC: negative control rats, NAC: N-acetylcysteine treated rats, CB-1-10: rats that are treated by test compounds, # - statistically reliable respecting the SO group of animals, * : statistically reliable respecting the NC of the rat group, α : statistically significant relative to the group of animals treated by N-acetylcysteine

When studying the effect of chromon-3-aldehyde derivatives on the change in the overall respirometric function of neuronal mitochondria in rats under conditions of cerebral ischemia, it was found that in NC group, in comparison with SO animals, ATP-generating ability, maximum respiration level and respiratory capacity decreased by 3.6 times (*P*<0.05); 11.9 times (*P*<0.05) and 7.7 times (*P*<0.05), respectively. At the same time, the administration of N-acetylcysteine contributed to an increase (in relation to the NC group of animals) of ATP-generating ability by 2.64 times (*P*<0.05); maximum respiratory rate - 8.04 times (*P*<0.05) and respiratory capacity - 4.42 times (*P*<0.05). Among the test objects, the most pronounced effect on the change in the overall respirometric function of mitochondria was exerted by the usage of a compound under a СВ-10 code against the background of which, an increase in ATP-generating ability, maximum respiration level and respiratory capacity compared to the same indices of NC rat group by 2.79 (*P*<0.05); 8.34 (*P*<0.05) and 4.77 times (*P*<0.05), respectively (Figure 4) was noted. When using the remaining studied derivatives of chromon-3-aldehyde (codes CB-1 - CB-9), an increase in ATP-generating ability, maximum respiration level and respiratory capacity (Figure 4) were also statistically significant in relation to the NC group of animals, however, observed the changes were not as pronounced as when the compound CB-10 was administered.

**Figure 5:**
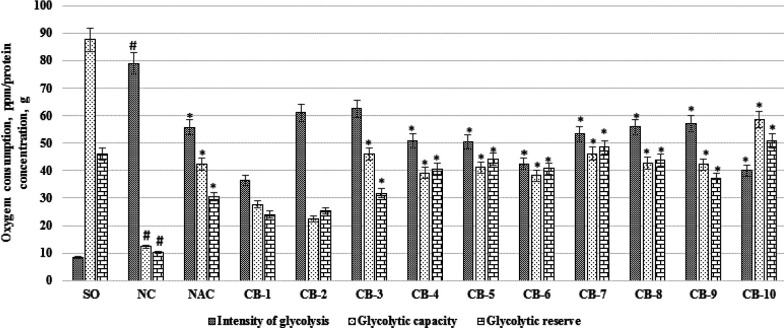
The effect of the test derivatives of chromon-3-aldehyde on the activity of anaerobic processes in brain neurons in rats under cerebral ischemia

Note: SO: sham-operated animals, NC: negative control rats, NAC: N-acetylcysteine treated rats, CB-1-10: rats that are treated by test compounds, # - statistically reliable respecting the SO group of animals, * : statistically reliable respecting the NC of the rat group, α : statistically significant relative to the group of animals treated by N-acetylcysteine

When assessing the effect of chromon-3-aldehyde derivatives on changes in the activity of anaerobic processes, an increase in the intensity of glycolysis by 9.56 times (*P*<0.05) in the NC group of rats relative to the SO group of animals was noted, accompanied by a decrease in glycolytic capacity and glycolytic reserve by 7, 08 times (*P*<0.05) and 4.49 times (*P*<0.05), respectively (Figure 5). Against the background of a N-acetylcysteine administration in rats, an increase in glycolytic capacity and glycolytic reserve was observed compared with the NC of the rat group by 3.42 times (*P*<0.05) and 2.98 times (*P*<0.05), respectively, with a decrease in the intensity of glycolysis by 29.54% (*P*<0.05). Among the test compounds, the most pronounced effect on the change in the activity of anaerobic processes was exerted by the maintenance of a chromon-3-aldehyde derivative under the code of CB-10, with the administration of which a decrease (in relation to the NC group of animals) of glycolysis intensity was noted - by 49.38% (*P*<0.05 ), as well as an increase in glycolytic capacity and glycolytic reserve by 4.74 times (*P*<0.05) and 4.95 times (*P*<0.05), respectively. Against the background of the use of other test substances CB-1; CB -4; CB -5; CB -6; CB 7; CB -8 and CB -9, the glycolysis intensity decreased in comparison with the NC group of rats by 53.8% (*P*<0.05); 35.7% (*P*<0.05); 36.1% (*P*<0.05); 46.3% (*P*<0.05); 32.5% (*P*<0.05); 29.3% (*P*<0.05) and 27.7% (*P*<0.05), respectively (Fig. 5). At the same time, when animals are administered compounds under the codes of CB-1; CB -4; CB -5; CB -6; CB 7; CB -8 and CB -9 showed a statistically significant (relative to the NC group of rats) increase in glycolytic reserve and glycolytic capacity, while the use of compounds CB -1 and CB -2 are not showed significant differences in the activity of anaerobic processes compared with the group of rats without pharmacological support (Figure 5).

***The effect of chromon-3-aldehyde derivatives on the change of the mitochondrial pore of transition permeability opening time***

**Figure 6.**
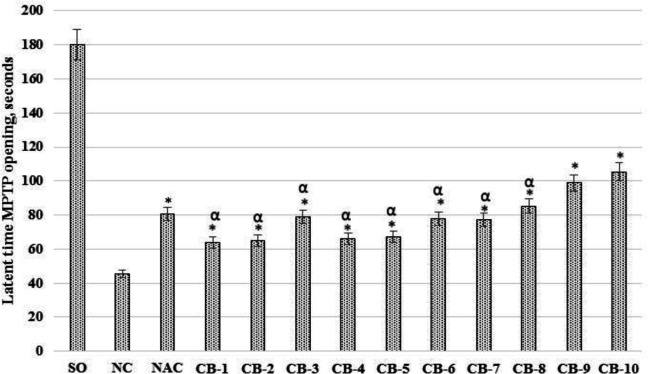
The effect of the test derivatives of chromon-3-aldehyde on the change in the opening time of the mitochondrial transition permeability pore in the neuronal mitochondria under cerebral ischemia in rats

Note: # : statistically reliable with respect to the SO group of animals, * : statistically reliable with respect to the NC rat group, α is statistically significant relative to the group of animals treated by CB-10 compound

In the course of this block of experimental work, it was found that the latent time of MPTP opening (Figure 6) was reduced by 3.9 (*P*<0.05) times in the NC group of rats compared with the SO group of animals. With the N-acetylcysteine use, an increase in the time required for the formation of MPTP in the neuronal mitochondria in rats was observed, in relation to the group of animals lacking pharmacological support by 77.7% (*P*<0.05). When using the test derivatives of chromon-3-aldehyde CB -1 - CB -10, an increase in the latent time of MPTP opening with respect to the same indicator of the NC of the rat group was observed. So, against the background of the introduction of compound CB -1, the time of formation of MPTP was less than that in the NC group of animals - by 41.2% (*P*<0.05); when using the compound CB -2 - 43.1% (*P*<0.05); CB -3 - 73.7% (*P*<0.05); CB -4 - 45.7% (*P*<0.05); CB -5 - 48.9% (*P*<0.05); CB -6 - 72% (*P*<0.05); CB 7 - 70.7% (*P*<0.05); CB -8 - 88.3% (*P*<0.05); CB -9 - 118.2% (*P*<0.05) and CB -10 - 132.6% (*P*<0.05). It should be noted that the most pronounced effect in the series of studied derivatives of chromon-3-aldehyde at the time of MPTP opening was exerted by the introduction of compound CB -10 (Figure 6).

***The effect of chromon-3-aldehyde derivatives on the change of the mitochondrial membrane potential value***

**Figure 7:**
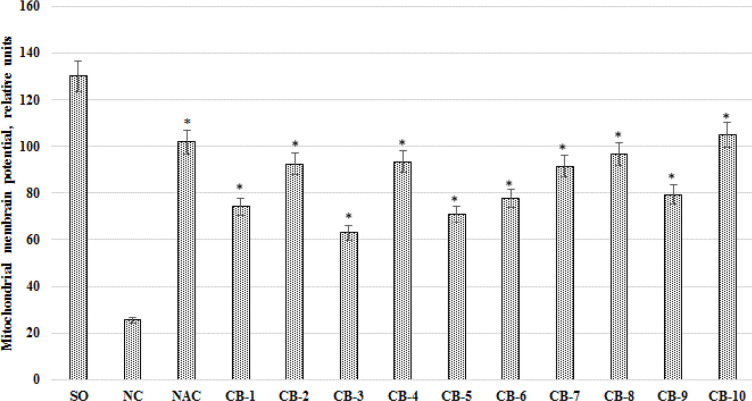
The effect of the test chromon-3-aldehyde derivatives on the change in the mitochondrial membrane potential of the neuronal mitochondria under cerebral ischemia in rats

Note: SO: sham-operated animals, NC: negative control rats, NAC: N-acetylcysteine treated rats, CB-1-10: rats that are treated by test compounds, # - statistically reliable respecting the SO group of animals, * : statistically reliable respecting the NC of the rat group, α : statistically significant relative to the group of animals treated by N-acetylcysteine

When assessing the effect of the test compounds on the change in the value of the mitochondrial membrane potential (Figure 7), it was found that in the group of rats without pharmacological support compared with the SO group, the value of the transmembrane mitochondrial potential decreased by 5.07 times (*P*<0.05). When using the reference drug, an increase in mitochondrial membrane potential was observed in comparison with the CK group of animals by 3.98 times (*P*<0.05). An increase in the transmembrane electronic gradient in the mitochondrial matrix was also noted against the background of the administration of the studied derivatives of chromon-3-aldehyde. So, the most significant changes were observed with the use of compound CB-1 (against the background of the administration of this test object, the value of the mitochondrial membrane potential increased by 4.1 times (*P*<0.05) compared with the NC group of rats). The use of substances under laboratory codes CB-1 - CB-9 contributed to an increase (relative to the NC group of animals) of the potential of the inner mitochondrial membrane by 2.89 times (*P*<0.05); 3.61 times (*P*<0.05); 2.46 times (*P*<0.05); 3.65 times (*P*<0.05); 2.77 times (*P* <0.05); 3.04 times (*P*<0.05); 3.58 times (*P*<0.05); 3.78 times (*P*<0.05) and 3.1 times (*P*<0.05), respectively (Figure 7).

***The effect of chromon-3-aldehyde derivatives on changes in the concentration of caspase-3 and apoptosis-inducing factor in rat brain neurons under cerebral ischemia***

**Figure 8:**
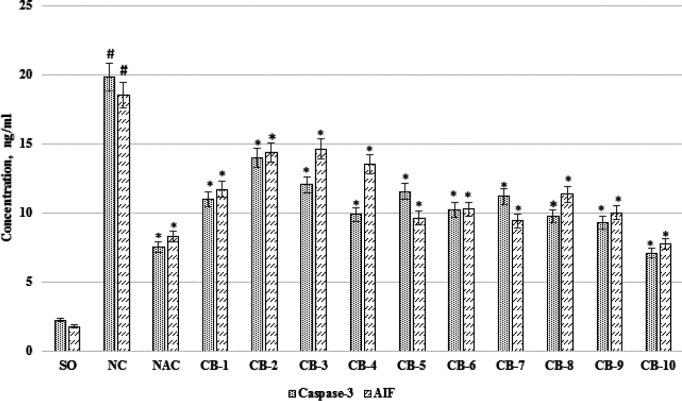
The effect of the test chromon-3-aldehyde derivatives on changes in the concentration of caspase-3 and AIF in the brain supernatant under cerebral ischemia in rats

Note: SO: sham-operated animals, NC: negative control rats, NAC: N-acetylcysteine treated rats, CB-1-10: rats that are treated by test compounds, # - statistically reliable respecting the SO group of animals, * : statistically reliable respecting the NC of the rat group, α : statistically significant relative to the group of animals treated by N-acetylcysteine

Assessing the change in the apoptotic processes activity in rat brain neurons under experimental cerebral ischemia, an increase in the concentration of caspase-3 and AIF (Figure 8) in NC groups of animals relative to SO rats by 8.9 (*P*<0.05) times and 10.4 (*P*<0.05) times, respectively was noted. At the same time, against the background of using the reference drug, a decrease in the content of proapoptotic markers in the supernatant of the animal brain - caspase -3 - by 62.2% (*P*<0.05) and AIF - by 55.3% (*P*<0.05) was observed. Among the test compounds, the most pronounced change in the activity of apoptotic systems was noted with the administration of the compound under the laboratory code CB-10. So, against the background of the administration of this chromon-3-aldehyde derivative to the animals, the concentration of caspase -3 and AIF decreased (compared to the NC group of animals) by 64.2% (*P*<0.05) and 58.2% (*P*<0.05) respectively. It should be noted that with the use of the rest of the test compounds, the content of caspase-3 and AIF relative to the group of rats lacking pharmacological support also decreased, however, the changes were not so pronounced as in the case of the administration of the substance under the CB-10 code (Figure 8).

## Discussion

Currently, mitochondrial dysfunction is recognized as one of the leading links in the «ischemic cascade» of brain damage (32). Mitochondria being «powefull stations» of a cell take part not only in the synthesis of macroergic compounds, but also regulate redox and apoptotic reactions in the cell. In recent decades, significant progress in mitochondrial medicine has been noted, largely related to the understanding of the effects of pharmacologically active compounds on mitochondria, their penetration into these cellular organelles and the identification of mitochondrial therapeutic «targets» (33). Also, the creation of new mitochondria-oriented compounds will significantly expand the range of selective drugs for the treatment of ischemic stroke (34). Nowdays, several types of pharmacologically active substances that can restore mitochondrial function are known: modified linker structures based on lipophilic cations (triphenylphosphonium, alkyl radicals) (35); mitochondria-oriented proteins (for example, SS series proteins - *Szeto-Schiller*) (34); biochemical shunting of mitochondrial complexes (succinic acid derivatives); antioxidants (N-acetylcysteine, tocopherols); energy-buffering agents (L-carnitine) (36). Also, one of the promising areas of pharmacological correction of mitochondrial dysfunction may be the use of compounds exhibiting electron donor-acceptor properties and capable of facilitating the electron transport reactions along the mitochondrial respiratory chain, for example, chromon derivatives having some structural similarity with coenzyme Q_10_. (37) At the same time, facilitating the movement of electrons along the respiratory chain probably helps to stabilize the mitochondrial respiratory potential, which ultimately increases the output of ATP (38).

In the study, it was found that the administration of chromon-3-aldehyde derivatives to animals contributed to the restoration of the functional activity of the mitochondrial respiratory chain, as evidenced by an increase in ATP-generating ability, maximum respiration level and respiratory capacity in animals treated by the test compounds, compared with not treated rats. Also, when using the test derivatives of chromon-3-aldehyde, a decrease in the intensity of anaerobic processes was noted, which can also be associated with a facilitation of electron transport from complex I to complex IV of the respiratory chain and an increase in the acceptor properties of oxygen. In addition, the stabilization of the activity of the mitochondrial respiratory chain observed at can positively affect the activation of apoptotic events (39). To date, it has been established that mitochondria play one of the key roles in the regulation of programmed cell death, participating in reactions of both the external and internal pathways of apoptosis. Moreover, the activation of the pro-apoptotic signal is largely associated with a deterioration in the energy-producing function of mitochondria, i.e. a decrease in the activity of mitochondrial complexes of the respiratory chain (40). One of the leading roles in this process is assigned to reducing the membrane potential of mitochondria and the formation of mitochondrial pores of transitional permeability (41). The high frequency of MPTP opening leads to calcium overload of the cells, with mitochondria swelling and, as a result, cytochrome C releasing increases with activation of caspase-dependent and caspase-independent apoptosis reactions (42). The study showed that the use of the test derivatives of chromon-3-aldehyde contributed to the stabilization of the mitochondrial membrane potential and a decrease in the rate of MPTP opening, which ultimately led to a decrease in the activity of capase-3, the main effector of the apoptosis external pathway (43) and AIF - the mitochondrial protein corresponding for the occurrence of caspase-independent apoptosis reactions (44). Ultimately, the change in mitochondrial function observed upon administration of chromon-3-aldehyde derivatives to animals contributed to a decrease of the brain necrosis area and neurological symptoms in rats with reproduced cerebral ischemia. It is worth noting that the test derivatives of chromon-3-aldehyde are low-toxic compounds, as the obtained LD_50_ value may indicate; however, it seems advisable to study the chronic toxicity of the studied compounds, which will make it possible to fully evaluate the toxicological profile of chromon-3-aldehyde derivatives. In addition, for some compounds with a chromon-3-aldehyde core in their structure, antioxidant properties are described, expressed in direct scavenger activity with respect to ROS (45) and an increase in the activity of endogenous antioxidant enzymes, such as superoxide dismutase, catalase, glutathione peroxidase (46). Moreover, antioxidant properties can also be an essential component of the positive effect of derivatives of chromon-3-aldehyde on the change of mitochondrial function.

In addition, in the work of Kawase *et al. *2007 provides data on the effect of chromon-3-aldehyde derivatives on the course of apoptotic reactions, while it was shown that bromine-substituted 3-formylchromone accelerates apoptosis in cancer cells, which may be associated with normalization of mitochondrial function and suppression of the Warburg effect (47). However, a study conducted by Sakagami, *et al. *2015, on the contrary, showed the presence of a toxic effect of chromon derivatives on the mitochondria. So in this work, obtained results indicating that the injection of chromon derivatives in HSC-2 cell culture contributed to the violation of the integrity of the outer membrane of mitochondria, their vacuolization and loss of functional activity. Probably the obtained data can be related to the structure of the test-substances – in the work of Sakagami*, et al. *2015 studied 6-methoxy-3-styryl substituted chromons (48). Also, a study by Zhang, *et al. *2014 shows modulating activity of chromon-3-aldehyde derivatives in relation to Bcl-2/Bax proteins, which may also affect changes in total mitochondrial function in this case, both an increase in mitochondrial function and its decrease are possible (49).

**Table 1:** Characteristics of the test-compounds

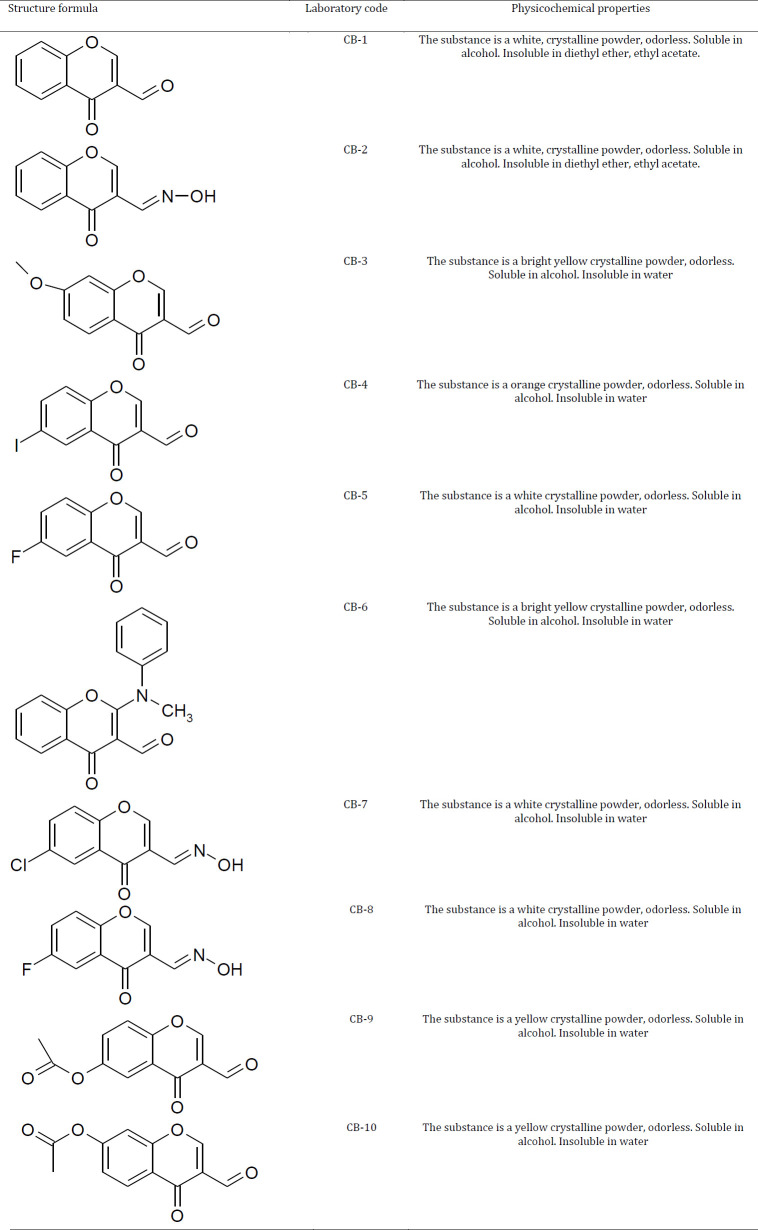

**Table 2:** McGraw neurological deficiency scale (1976)

Evaluated parameters	Neurological deficit score
Inactivity	0.5
Tremor	1
Hemiptosis* by one-side	1
Double-side hemiptosis*	1.5
Limb feebleness	1.5
Ptosis by one-side	1.5
Ptosis by double-side	1.5
Circular motion	2.0
Limbs paresis (1-4)	2–5
Limb paralysis (1-4)	3–6
Coma	7.0
Death	10.0

**Table 3:** The results of the evaluation of acute toxicity chromon-3-aldehyde derivatives

**Test compound**	**LD**_50_ ** mg / kg, orally**	**The administered dose of mg/kg (** ***per os*** **), for further research (1/100 of LD**_50_ **)**
СВ-1	3361±562.14	33.61
СВ-2	2763±962.63	27.63
СВ-3	3262±451.63	32.62
СВ-4	3826±751.25	38.26
СВ-5	6131±336.98	61.31
СВ-6	2831±228.94	28.31
СВ-7	2645±592.31	26.45
СВ-8	2408±761.94	24.08
СВ-9	2930±265.45	29.3
СВ-10	4000±175.91	40

## Conclusion

Based on the obtained data, it can be assumed that the derivatives of chromon-3-aldehyde have cerebroprotective activity, expressed in a decrease of the cerebral infarction area and neurological deficit in rats on the model of irreversible thermocoagulation of the middle cerebral artery. Moreover, probably the cerebrotropic properties of the chromon-3-aldehyde derivatives be associated with the complex effect of these compounds on the change in mitochondrial function, expressed in the stabilization of electron transport reactions along the mitochondrial respiratory chain and mitochondrial membrane potential, as well as a decrease in the rate of opening of the mitochondrial pore of transition permeability and the intensity of apoptotic reactions. The obtained results can be the basis for further deeper study of the pharmacological activity and toxicological properties of chromon-3-aldehyde derivatives.
